# Role of Vitamin D on the Inhibition of Gastrin Production After Cisplatin Treatment

**DOI:** 10.1155/MBD.2000.115

**Published:** 2000

**Authors:** Ying Wang, Surinder K. Aggarwal, Will Kopachik

**Affiliations:** Department of Zoology Michigan State University East Lansing Michigan 48824-1115 USA

## Abstract

In rats cisplatin induces hypocalcemia, bloating of the stomach, and ulceration ameliorated through calcium supplements. This study was undertaken to test the role of calcium on the gastrin mRNA production *in vitro* and *in vivo*. RIN B6 cells were cultured in medium with calcium (1.8, 3.6 and 7.2 mM) and the active form of vitamin D (calcijex). Cisplatin was added (10 μg/ml) for 12 hrs and cells were harvested for RNA from various treatment groups. Male Wistar rats were treated with cisplatin (9 mg/kg), before and after vitamin D (0.3 mg/100g/week). The rats were killed and stomach tissues excised on 1, 6, 10 and 15 days after cisplatin treatment. RNA from the stomach was analyzed using the northern blot technique. Gastrin mRNA was suppressed after cisplatin treatment both *in vitro* and *in vivo*. *In vitro* calcium but not vitamin D additions partially prevented the gastrin mRNA. *In vivo*, however, vitamin D and calcium were equally effective in preventing gastrin mRNA loss.


### ROLE OF VITAMIN D ON THE INHIBITION OF GASTRIN PRODUCTION AFTER CISPLATIN TREATMENT

Ying Wang, Surinder K. Aggarwal* and Will Kopachik

Department of Zoology, Michigan State University, East Lansing, Michigan, U.S.A. 48824-1115

**Abstract**

In rats cisplatin induces hypocalcemia, bloating of the stomach, and ulceration ameliorated through calcium supplements. This study was undertaken to test the role of calcium on the gastrin mRNA production *in vitro* and *in vivo*. RIN B6 cells were cultured in medium with calcium (1.8, 3.6 and 7.2 mM) and the active form of vitamin D (calcijex). Cisplatin was added (10 µg/ml) for 12 hrs and cells were harvested for RNA from various treatment groups. Male Wistar rats were treated with cisplatin (9 mg/kg), before and after vitamin D (0.3 mg/100g/week). The rats were killed and stomach tissues excised on 1, 6, 10 and 15 days after cisplatin treatment. RNA from the stomach was analyzed using the northern blot technique. Gastrin mRNA was suppressed after cisplatin treatment both *in vitro* and *in vivo*. *In vitro* calcium but not vitamin D additions partially prevented the gastrin mRNA. *In vivo*, however, vitamin D and calcium were equally effective in preventing gastrin mRNA loss.

### Introduction

Cisplatin (cis-dichlorodiammineplatinum II; CDDP), a broad-spectrum anticancer drug, is effective in the treatment of a variety of cancers. One drawback is severe toxic side effects including nausea, vomiting, and the induction of peptic ulcers in the gastrointestinal tract [1]. Ulceration of the stomach is due to the erosive acid/peptic factors, which exist in normal stomach content [2]. Both exogenous and endogenous gastrinshow a gastroprotective effect by maintenance of gastric mucosal integrity [3, 4]. There is however, a significant suppression of gastrin production after cisplatin treatment, suggesting gastrin loss might be one factor leading to ulceration [5].

In rats given cisplatin a lack of vomiting reflexes, causes bloating of the stomach and ulceration [1]. The bloating of stomach is associated with hypocalcemia due to cisplatin [6]. Calcium plays a role in release of acetylcholine from the nerve fibers, inducing relaxation of the pyloric sphincter and contraction of the gastric smooth muscles [1, 7]. *In vitro*, fundal strips from cisplatin-treated rats are hypercontractile to acetylcholine in calcium-free Tyrode solution, but contract normally in Tyrode solution with calcium. Thus there is a clear role for calcium in cisplatin-treated smooth muscle contraction[8]. Pretreatment with calcium prevents the bloating of the stomach due to cisplatin [1, 9]. Hypocalcemia is also associated with inhibition of ATP synthesis and various membrane transport enzymes. Again pretreatment with calcium has a protective effect on these enzymes [6, 9].

The present study was undertaken to test for a direct role of vitamin D in cell cultures in comparison to the indirect effect through calcium homeostasis in the rats.

### Materials and Methods

### *Cell culture*

RIN B6 cells (rat insulinoma cell line)[10], a generous gift from Dr. Loyal Tillotson, Department of Medicine, University of North Carolina, were cultured at 37°C in Dulbecco's modified Eagle's medium (DMEM): 4.5 g/ml glucose with 10% fetal bovine serum, 100 mg/ml streptomycin and 100 units/ml penicillin in an atmosphere of 5% $CO_2$. Calcium chloride stock solution (1 M) was added to achieve the calcium concentrations of 3.6 mM or 7.2 mM. EDTA (1.5mM) was used to chelate the calcium in the culture medium. Some cultures were treated with calcijex (1,25-dihydrox-Vitamin $D_3$, from Abbott Laboratories, Chicago, IL) at a 50 pg/ml concentration, calcijex plus calcium (3.6 mM), or calcijex plus calcium and cisplatin (10µg/ml). Cells were also cultured in cisplatin containing medium with calcijex or calcium alone. Untreated cultures served as controls. Cisplatin (10µg/ml) was added to various cultures for 12 h. The cells were trypsinized and stained with trypan blue (GIBCO BRL, Grand Island, NY). Cell counts were taken and RNA was extracted in all the experiments by methods previously described [11]

*Animals and Tissue Preparation*

Male Wistar rats (Charles River Laboratory; Wilmington, MA) weighing 100-150 g were kept in a 12-h light/12-h dark cycle with free access to food and water. The rats were divided into 3 groups. The first group of rats was injected (IP) with cisplatin (9 mg/kg), in 0.9% normal physiological saline in five divided doses over 5 days. Rats in the second group were given vitamin D (0.3 mg/100g) (Banner Pharmacaps Inc., Elizabeth, NJ) once a week for the duration of the experiment. And given cisplatin (9 mg/kg) after one week after the first vitamin D injection. The third group of rats received the injection of vehicle only. Three rats from each group were sacrificed and stomach tissues excised 1, 6, 10 and 15 days afterof the last cisplatin injection and frozen (-70°C) until use. The experiment was repeated at least 3 times.

*Northern blot analysis*

A 27-mer sequence 5'-GACCTTGGGGCCCCAGCTGTCTCCGAT-3', complementary to the coding sequence of rat gastrin mRNA (position 212-238) [12], was synthesized using the ABI 3948 Synthesis and Purification System. The gastrin oligonucleotide probe was labeled with the digoxigenin (DIG) oligonucleotide tailing kit (Boehringer Mannheim Corp.Indianapolis, IN). A 100 pmol probe was incubated with tailing buffer, $CoCl_2$ solution, DIG-dUTP, dATP and terminal transferase solutions at 37°C for 18 minutes, then placed on ice and the reaction was stopped by adding 4μl stop solution. The 28s rRNA probe was labeled using DIG high prime kit (Boehringer Mannheim Corp. Indianapolis, IN). The rRNA probe (50ng) in 16 μl water was denatured and incubated with 4 μl DIG high prime mix for 20 hours. The reaction was stopped by adding 2 μl 0.5 M EDTA. The labeled probes were precipitated in 5μl 4M LiCl and 150 μl prechilled (-20°C) ethanol at –20°C for 3 hours and then centrifuged at 12,000 g for 15 minutes. The supernatant was discarded and the pellet was air-dried.

RNA was extracted from the whole stomach tissues or cells by guanidinium isothiocyanate solution and purified through a CsCl cushion[11]. The RNA (20 μg) was size-separated by electrophoresis in a 1.3% agarose gel containing formaldehyde and electroblotted onto nylon membrane (GeneScreen, New England Nuclear, Boston, MA). The blots were prehybridized with the buffer containing 50% formamide, 10% dextran sulfate, 50 mM Tris (pH 6.8), 3X standard saline citrate (SSC), 100 mg/ml sonicated salmon sperm DNA and 5X Denhardt's solution for 3 hours at 42°C. Blots were then incubated with fresh hybridization buffer in the presence of labeled probes, 40 ng/ml for gastrin and 5 ng/ml for 28s rRNA, at 42°C for 2 days. The blots were washed with two changes of 2X SSC/0.1% sodium dodecyl sulfate (SDS) at 42°C for 30 min each and 0.1X SSC/0.1% SDS at 42°C for another15 min. The DIG in the probes was detected using a DIG nucleic acid detection kit (Boehringer Mannheim Corp. Indianapolis, IN). The blots were washed in maleic acid buffer (pH 7.4) for 10 min, then sequentially incubated in blocking buffer for 30 min and followed by 1/1000 anti-DIG antibody for 45 min. After washes in PBS and 100mM Tris-HCl buffer (pH 9.5), the blots were incubated in nitroblue tetrazolium-bromochloroindolyl phosphate (NBT/BCIP) solution overnight. RNA extracts from liver tissue served as control for the absence of gastrin. All the $H_2O$ used in the above study was diethyl pyrocarbonate (DEPC) treated double distilled water.

*Statistical Analysis*

The number of viable versus dead cells from cultures with different concentrations of calcium, with or without cisplatin and/or calcijex were counted. The data was statistically analyzed by the One-way ANOVA test [13].

### Results

RNA extracted from RIN B6 cells was subjected to electrophoresis and hybridization with DIG labeled probe, specific for gastrin mRNA (Fig. 1). In the absence of calcium in the culture medium, the cells had negligible levels of gastrin mRNA. However, when supplemented with calcium (1.8mM) the gastrin mRNA levels were equivalent to those in normal stomach tissue (Fig 1). Cisplatin treatment of RIN B6 cells in presence of calcium (1.8 mM) significantly inhibited gastrin mRNA levels. Cisplatin treatment, however, with 3.6 mM or 7.2 mM of calcium restored near normal levels of gastrin mRNA. Vitamin D supplementation did not affect the control of gastrin mRNA level (Fig. 2/ lanes 2 and 7). Cisplatin treatment of RIN B6 cells inhibited gastrin mRNA levels with or without vitamin D (lanes 5 and 6). In contrast calcium supplementation maintained the gastrin mRNA levels in both cell cultures with or without vitamin D (lanes 3 and 8). In order to monitor the amount and integrity of RNA sample loading in each lane, rRNA (28s) served as a control. The rat stomach RNA served as a positive control for gastrin mRNA, whereas liver RNA served as a negative control (data not shown).

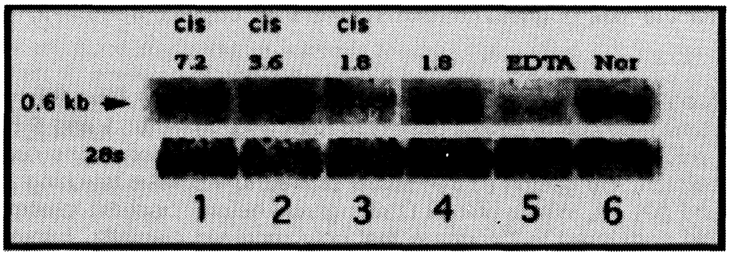

Figure 1. Northern blot analysis of gastrin mRNA from the RIN B6 cells cultured in different concentrations of calcium with or without cisplatin. Note the negligible levels of gastrin mRNA in the absence of calcium (EDTA) from the culture medium (lane 5). After supplementing calcium (1.8mM) the gastrin mRNA levels were maintained to normal (lane 4). Cisplatin treatment inhibited gastrin mRNA production (lane 3), however, in the presence of 3.6 mM or 7.2 mM of calcium gastrin mRNA levels were close to normal (lanes 2, 1). Gastrin mRNA from the rat stomach served as a molecular size marker for gastrin mRNA. Equal amounts of 28s ribosomal RNA were detected in all samples.

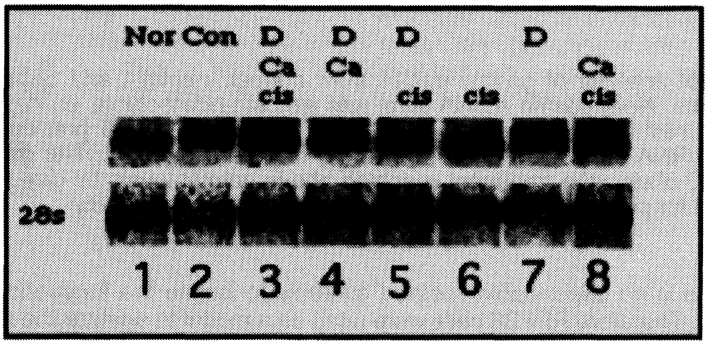

Figure 2. Northern blot analysis of gastrin mRNA from the RIN B6 cells showing the effects of calcium and/or vitamin D on the inhibition of gastrin production after cisplatin administration. Note the suppression of gastrin mRNA after cisplatin (lane 6) or cisplatin plus calcijex (lane 5) treatments. Calcijex by itself has no effect (lane 7). However, calcium (3.6mM) seems to counteract the suppression of gastrin mRNA after cisplatin treatment (lane 8), as does calcijex and calcium (lane 3). Gastrin mRNA from the rat stomach served as a molecular size for gastrin mRNA (lane 1). Equal ribosomal RNA (28s) was detectable in all samples.

Table 1. Number of viable and dead cells from cultures with different concentrations of calcium, with or without cisplatin and/or calcijex. (p=0.27)

| | | | | | | | | |
|---|---|---|---|---|---|---|---|---|
| Calcium concentration (mM) | 1.8 | 3.6 | 3.6 | 1.8 | 1.8 | 1.8 | 3.6 | 7.2 |
| Cisplatin (10 μg/ml) | – | + | – | + | + | – | + | + |
| Calcijex (50pg/ml) | – | + | + | + | – | + | – | – |
| Time of cisplatin treatment (hr) | 0 | 12 | 12 | 12 | 12 | 12 | 12 | 12 |
| Viable cells ($10^6$/ml) | 4.6 | 4.7 | 4.4 | 3.4 | 3.6 | 3.2 | 3.9 | 3.3 |
| Dead cells ($10^5$/ml) | 2.6 | 2.7 | 2.5 | 2.7 | 1.4 | 1.7 | 1.5 | 1.8 |
| Percentage of dead cells (%) | 5.7 | 5.4 | 5.7 | 7.9 | 3.9 | 5.3 | 3.8 | 5.1 |

+, treatment; --, no treatment

To ensure that cell viability was unaffected by the various treatment regimes the percentage dead cells was determined (Table I). We conclude that there are insignificant differences in cell viability which could account for the loss of gastrin mRNA in cells after cisplatin treatment. The cell counts are depicted in Table 1.

Rats were treated with cisplatin and cisplatin plus vitamin D. RNA from the rat stomach tissues was analyzed by the same methods to detect gastrin m RNA (Fig. 3). After 1 and 6 days since cisplatin treatment, the gastrin mRNA bands were almost undetectable, compared to the control. However, 10 days after cisplatin treatment the gastrin mRNA levels started to increase reaching levels found in the untreated animal around day 15. When vitamin D was given before cisplatin treatment gastrin mRNA levels were not reduced compared to the group that only received cisplatin. Interestingly the gastrin mRNA increased to greater levels by day 15. The equal amount of loading was shown by the equal intensity of the 28s bands.

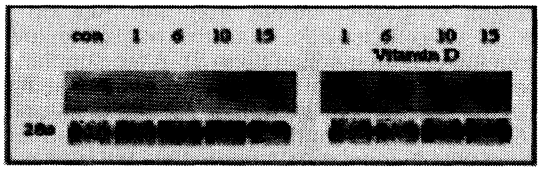

Figure 3. Northern blot analysis of gastrin mRNA from normal, cisplatin and cisplatin plus vitamin D treated animal stomachs. Note gastrin mRNA level was almost undetectable on day 1 and day 6 after cisplatin treatment but restored to normal levels at day 10.. In the animals pretreated with vitamin D, however, the gastrin mRNA levels were not lower after cisplatin treatment. The gastrin mRNA started to increase after day 10 of cisplatin treatment reaching above normal levels by day 15. Ribosomal RNA (28s) served as control for possible variation in sample RNA amount or integrity.

### Discussion

Currentlu there is no dependable method available to isolate the functional gastrin-producing cells from the stomach. Therefore RIN B6 cells were used as a model to analyze the regulation of gastrin gene expression [10]. Although EDTA treatment inhibited the expression of gastrin mRNA, exogenous calcium restored it to normal levels [10]. Higher levels of extracellular calcium might be expected to increase the amount of calcium transported through the calcium channels into the cell. Gastrin gene expression may be maintained in the RIN B6 cells by elevation of intracellular calcium [10]. The mechanism of how cisplatin decreases, and calcium increases gastrin mRNA levels is unknown. Calcium may counteract lowered transcription rates or increased mRNA degredation.. Cisplatin has been shown to bind covalently with DNA in intra- and inter-strand crosslinks preventing its replication or transcription [14]. In order for us to interpret the decrease in the gastrin mRNA levels in cultures specifically due to cisplatin induced cell death, we counted the number of dead cells after each treatment. Our results documented no significant changes in number of viable cells amongst different treatments. Therefore, we infer that decreased levels of gastrin mRNA are not due to the loss of cells. Vitamin D, in order to be effective as a regulator of calcium homeostasis, is hydrolyzed into an active form of vitamin D (1,25-dihydrox-Vitamin $D_3$) in the liver and kidney. The active form of vitamin D did not show any demonstrable changes in the mRNA levels of gastrin. Vitamin D acts via a nuclear receptor to affect gene transcription[16] It is conceivable that the RIN B6 cells lack this receptor and therefore fail to respond. Alternatively vitamin D may act by increasing calcium only *in vivo*. However, exogenous calcium demonstrated a profound protective effect on gastrin mRNA after cisplatin treatments. In support of this mechanism cisplatin treatments have been shown to cause severe hypocalcemia in rats [1, 6]. However, pretreatment with vitamin D is able to maintain the serum calcium levels within the normal range [15]. In vitro, vitamin D or calcijex did not show any protective effect against cisplatin suppression of gastrin mRNA, however, calcium supplement demonstrated a significant protective effect. In vivo, vitamin D was just as effective in maintaining the gastrin mRNA levels possibly by prevention of hypocalcemia.

**Acknowledgment**

This research was supported by a Student Award Program Grant from the Blue Cross Blue Shield of Michigan Foundation (352-SAP/99).

**Reference**

1 Aggarwal S K, Antonio J D S, Sokhansanj A and Miller C M. *Anti-Cancer Drugs* 1994; **5**:177.
2 Mertz H R and Walsh J H. *Med Clin North Am* 1991; **75**:799.
3 Stroff T, Plate S, Respondek M, Muller K M and Peskar B M. *Gastroenterology* 1995; **109**:89.
4 Konturek S J, Brzozowski T, Bielanski W and Schally A V. *Eur J Pharmacol* 1995; **278**:203.
5 Wang Y, Aggarwal S K and Painter C L. *J Histochem Cytochem* 1999; **47**:1057.
6 Aggarwal S K. *J Histochem Cytochem* 1993; **41**:1053.
7 Jarve R K and Aggarwal S K. *Cancer Chemother Pharmacol* 1997; **39**:341.
8 San Antonio J D and Aggarwal S K. *J Clin Hematol Oncol* 1984; **14**:55.
9 Aggarwal S K and Fadool J M. *Anti-Cancer Drugs* 1993; **4**:149.
10 Brand S J and Wang T C. *J Biol Chem* 1988; **263**:16597.
11 Chirgwin J M, Przybyla A E, MacDonald R J and Rutter W J. *Biochemistry* 1979; **18**:5294.
12 Larsson L I and Hougaard D M. *J Histochem Cytochem* 1993; **41**:157.
13 Campfield T, Braden G, Flynn-Valone P and Powell S. *Pediatrics* 1997; **99**:814.
14 Andrews P and Howell S. *Cancer Cells* 1990; **2**:35.
15 Brown A J, Dusso A and Slatopolsky E. *Am J Physiol* 1999; **277**:F157.
16. Minghetti P P and Norman A W. *FASEB J* 1988; **2**: 3043.

**Received: February 18, 2000 - Accepted: March 10, 2000 - Received in publishable format: April 25, 2000**

